# Rare genetic Creutzfeldt-Jakob disease with E196A mutation: a case report

**DOI:** 10.1080/19336896.2019.1631679

**Published:** 2019-06-25

**Authors:** Yanyuan Dai, Yue Lang, Mingxuan Ding, Baizhuo Zhang, Xiaoou Han, Guangyu Duan, Li Cui

**Affiliations:** Department of Neurology, Neuroscience Center, The First Hospital of Jilin University, Jilin University, Changchun, China

**Keywords:** Genetic Creutzfeldt-Jakob disease, PRNP, mutation, E196A

## Abstract

Genetic Creutzfeldt-Jakob disease (gCJD) accounts for approximately 10–15% of human prion diseases. It is an autosomal dominant disease caused by missense or insertion mutations of the gene that encodes prion protein (PRNP). In general, the manifestations and neuropathological changes of gCJD are similar to those of sporadic CJD (sCJD), and the diagnostic sensitivities of cerebrospinal fluid (CSF) markers, electroencephalography (EEG), and magnetic resonance imaging (MRI) are generally lower in gCJD than sCJD. Here we report on a 56-year-old Chinese woman who was diagnosed with gCJD and suspected to have thyroid cancer. The patient carried the glutamate to alanine substitution at codon 196 (E196A) of PRNP, which is quite a rare mutation and has only been reported in China. To our knowledge, this is the fourth case of E196A gCJD in the world. Here, we compared the manifestations and assistant examinations of the current patient with those of three previously reported Chinese patients with E196A gCJD in order to illustrate the common features of E196A gCJD.

## Introduction

Human prion disease, or transmissible spongiform encephalopathy (TSE), is a rare, transmissible, fatal, and rapidly progressive neurodegenerative disease caused by the expansion and accumulation of misfolded prion protein (PrP^sc^) in the central nervous system. TSE is divided into three subtypes according to the aetiology of the disease: sporadic, genetic, and acquired. Sporadic Creutzfeldt-Jakob disease (sCJD) is the most common type of TSE and accounts for about 80% to 95% of all cases. Genetic TSE, including genetic Creutzfeldt-Jakob disease (gCJD), Gerstmann–Straussler–Scheinker syndrome (GSS), and fatal familial insomnia (FFI), accounts for approximately 10–15% of all cases. Acquired TSE includes iatrogenic Creutzfeldt-Jakob disease (iCJD) and variant Creutzfeldt-Jakob disease (vCJD), accounts for 1% of all cases [1,2].

Genetic prion diseases (gPrDs) are caused by point or insertion mutations in the prion protein (PrP) encoding gene, PRNP, which is the only gene known to be associated with gPrDs [3]. The PRNP mutation is necessary for the diagnosis of gCJD in symptomatic individuals [1]. PRNP is located on the short arm of chromosome 20. The cellular prion protein (PrP^c^) encoded by PRNP is composed of 253 amino acids before post-translation modification, and its mature form is a peptide that is composed of 208 amino acids that is fixed on the cell membrane in a unique way [4]. To date, more than 60 mutations have been identified in the PRNP open reading framework; more than 20 of these mutations are associated with gCJD [5]. The manifestations of gCJD are mainly characterized by rapidly progressive cognitive decline (mainly manifested as memory loss), visual or cerebellar problems, myoclonic jerks, pyramidal or extrapyramidal features, and akinetic mutism [6]. Additionally, the age of onset of gCJD (mean age, 30–55 year of age) occurs at a correspondingly younger age than sCJD (55–75 years old), and the duration of gCJD is comparatively longer than sCJD (usually <1 year) [7]. However, different clinical phenotypes are observed in patients with gPrDs depending on differences in gene mutations and PRNP polymorphisms of codon 129, which encodes methionine (M) or valine (V) [7]. PRNP mutations are geographically and ethnically distinct, and clusters of gPrDs have been observed in some regions of Israel, Slovakia, Chile, and Italy [8]. E200K is the most common PRNP mutation, and E200K gCJD is the most common gCJD worldwide. However, T188K gCJD is most frequently identified in the Chinese population [8]. This report described a newly identified patient who was diagnosed with gCJD and carried a point mutation at codon 196 (E196A: GAG-GCG). To our knowledge, E196A gCJD is very rare and has only been reported in China [9]. Including the current patient, only four cases of E196A gCJD have been reported worldwide.

## Case report

A retired 56-year-old female was admitted to our hospital owing to a progressive movement disorder. Before admission, the patient reported numbness and weakness of the left limbs, gait difficulties, obvious dizziness, and paroxysmal tremor of the left limbs for almost 50 days. Difficulties in the movement of her left limbs gradually worsened. Upon admission, the patient began to have involuntary flexion in the distal left extremities but had no obvious cognitive impairment. Evaluations for amyotrophic lateral sclerosis, other motor neuron diseases, Parkinson’s disease, and other movement disorders were negative. She had no relevant family history and no exposure to neurotoxins, biologic agents, ticks, or rabies. However, we suspected carcinoma of the right thyroid lobe after the pathological analysis of the thyroid. Neurologic examination revealed temporal hemianopia in the left eye, an increase in the muscle tone of the left extremity, and hyperreflexia, which was suggestive of higher cortical dysfunction. Cerebellar tests were hard to assess because of the patient’s tremor. The sensory examination showed no obvious abnormalities. There are no signs of meningeal irritation to suggest mening involvement. The electromyography method could not be conducted because the patient was unable to cooperate. During hospitalization, the patient showed progressive cognitive decline and gradually increased muscle tone. At 2 months post-hospitalization, the patient’s symptoms aggravated rapidly, and she presented with frequent myoclonic jerks and apparent psychotic symptoms, which included dreaminess, anxiety, and hallucinations during the night. The following week, the patient showed akinetic mutism.

**Figure 1.**
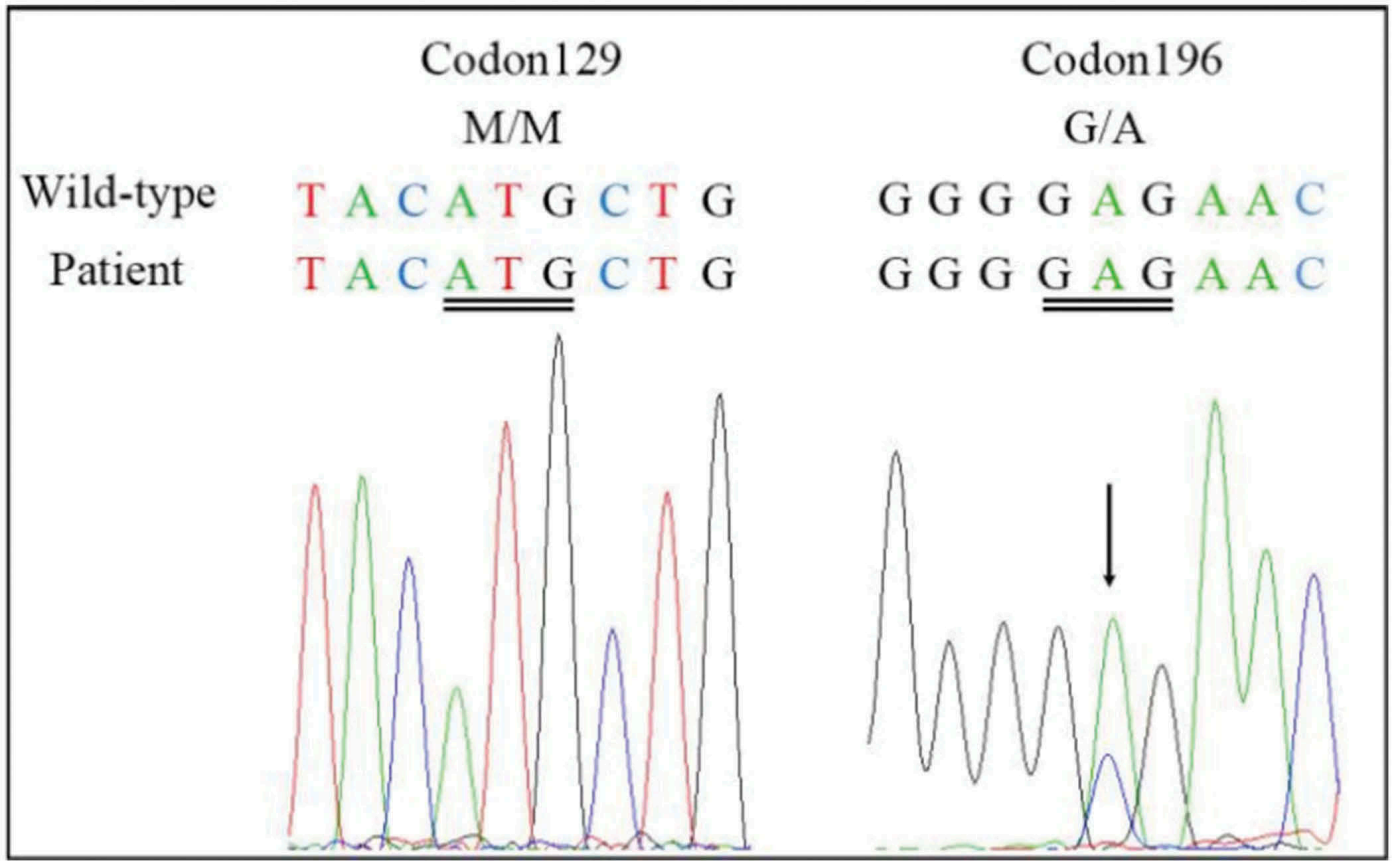
DNA sequencing of the PRNP gene. Compared with the standard sequence, the substitution of cytosine with adenine at codon 196 of the PRNP gene results in the substitution of alanine for glutamate (E196A). The PRNP polymorphism of codon 129 was 129M/M homozygous.

**Figure 2.**
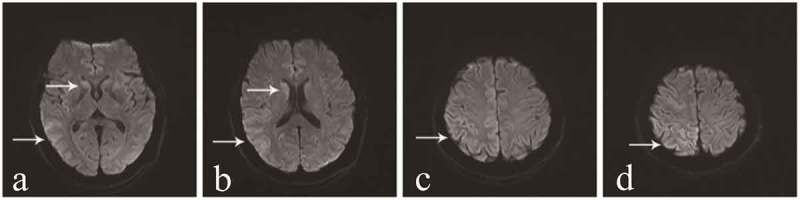
Results of DWI scans. **a ** and **b ** show an increase in signal intensity changes at the surface of the temporal lobe and bilateral basal ganglia regions (especially the caudate nucleus). **c ** and **d ** show ribbon-like signals in the right parietal lobe.

**Figure 3.**
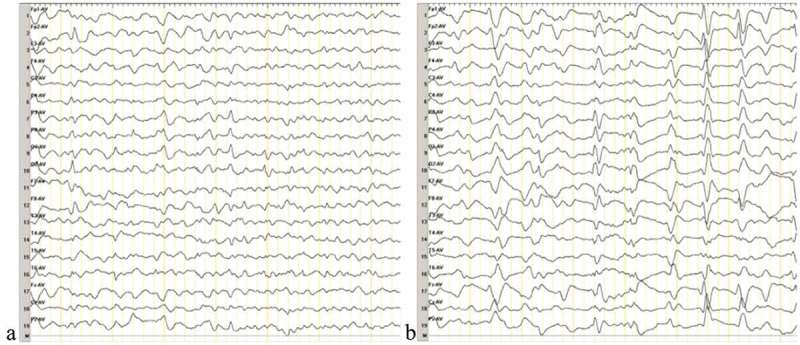
Results of the second EEG. The second EEG was performed at 2 months after onset of the movement disorder, and a week after the first EEG. The results of the two EEG examinations were similar. **a ** demonstrates abnormal waves in the background. **b ** displays sharp waves, sharp slow waves, and slow waves that are discharged synchronously or asynchronously at the bilateral occipital and frontal region.

The differential diagnosis included various dementing disorders, and comprehensive assessments, including neurophysiologic tests and multiple cerebrospinal fluid (CSF) and blood analyses, were performed. Following genetic testing, the patient was diagnosed with gCJD. As compared to the standard PRNP sequence (NCBI: NM183079.1), the sequence analysis of the PRNP gene in the current patient showed a missense mutation occurring at codon 196 (E196A: GAG-GCG) (Figure 1). The 14–3-3 protein in CSF was positive. The level of neuron-specific enolase (NSE) in blood was higher (45.45 ng/ml,<25.00 ng/ml). The autoimmune encephalitis-related antibodies in CSF were negative. In the current case, the diffusion-weighted magnetic resonance imaging (DWI/MRI) showed an increase in signal intensity changes at the surfaces of the right temporal and parietal lobes, left parietal lobe, and the bilateral basal ganglia region (Figure 2). Electroencephalography (EEG) was performed twice, but no typical periodic sharp wave complexes (PSWCs) were observed (Figure 3). The patient’s relatives declined a brain biopsy and autopsy; therefore, the present case lacked neuropathological data. The pathological analysis of the thyroid showed atypical degenerative cells in the right lobe of the thyroid, which was indicative of thyroid cancer.

## Discussion

To date, there have only been four E196A gCJD cases reported worldwide, and all of these cases have been diagnosed in China [10]. Patient characteristics are summarized in Table 1. There was no familial history of CJD among the four patients diagnosed with E196A gCJD. Three of the patients were women in their 50s, and the other patient was a man in his late 70s. The manifestations of these patients were similar to the symptoms associated with sCJD, including rapid, progressive cognitive decline (RPD); myoclonus; pyramidal and extrapyramidal symptoms; and a state of akinetic mutism during the late stage of the disease. In addition, the four patients showed obvious psychiatric symptoms and visual and cerebellar problems. The initial symptoms of the previously patients were different, two of them onset with intelligence decline, the other onset with dysarthria. Interestingly, numbness and involuntary tremor of the limbs were the initial symptoms reported by the patient in the current case, and a significant decline in cognitive function was not observed in the early stage of this patient. These were different from the other three cases. Moreover, at 2 months after the onset of the movement disorder, the patient showed a state of akinetic mutism, which indicated that the disease was rapidly progressing. The PRNP codon 129 genotype polymorphism was M/M in the four patients with E196A gCJD., and the 14–3-3 protein was positive in the CSF. In the current case, the DWI/MRI showed ribbon-like signals in the cortex and an increased signal intensity in the bilateral basal ganglia regions, especially the caudate nucleus. The similar DWI abnormalities were noticed in all previously patients. The classic PSWCs were only observed in the EEG of case 1.

**Table 1.** Clinical characteristics of the four patients with E196A gCJD.

	Case 1	Case 2	Case 3	Case 4
Age at onset	76	55	57	56
Gender	Male	Female	Female	Female
Duration	10	22	NA	NA
Familial history	-	-	-	-
Initial symptoms	Intellectual decline, and intermittent mental and behavioral disorders	Persistent decline in intelligence, giddiness, and unsteady walk	Dysarthria and weakness of the left hand	Numbness and weakness of the left limbs, obvious dizziness, and paroxysmal jitter of the left limb
RPD	+	+	+	+
Myoclonus	+	+	+	+
Visual problem	+	+	+	+
Cerebellar problems	+	+	+	-
Pyramidal symptoms	+	+	+	+
Extrapyramidal symptoms	+	+	+	+
Akinetic mutism	+	+	+	+
Psychiatric symptoms	+	+	+	+
14–3-3 Protein in CSF	+	+	+	+
MRI(DWI) increased signal intensity changes in the caudate or putamen)	-	+	+	+
MRI(DWI) ribbon-like signals)	+	-	+	+
PSWCs in EEG	+	-	-	-
Other EEG features	-	Bilateral diffuse waves, occasionally with periodic sharp waves	Moderate abnormality especially in the right hemisphere	A lot of abnormal waves in the background, including sharp waves, sharp slow waves and slow waves
Codon 129	MM	MM	MM	MM

**Abbreviation**: NA, not available; RPD, rapidly progressive dementia; CSF, cerebrospinal fluid; MRI, magnetic resonance imaging; DWI, diffusion-weighted imaging; PSWCs, periodic sharp wave complexes MM, methionine-methionine

The pathological changes that are associated with gCJD include diffusely distributed spongiform change, astrogliosis, and neuronal loss. Amyloid plaque deposition is not commonly observed in patients with gCJD. Differences in the neuropathology of various cases of gCJD are likely associated with the type of PrP^SC^ and polymorphisms in codon 129 [11].

There is a well-known non-pathogenic polymorphism in the PRNP gene (M/M、M/V、V/V) at codon 129. It has long been recognized that 129M/M homozygotes are overrepresented in sCJD and vCJD patients [12]. However, animal experiments have not yet evaluated the relationship between the PRNP polymorphism of codon 129 and the susceptibility of gPrDs. Through retrospective analyses, an overrepresentation of the 129M/M genotype and underrepresentation of the 129M/V genotype have been recognized in patients with E200K gCJD, V210I gCJD, P102L GSS, and D178N FFI [13]. In Japan, 129M/V heterozygotes have been overrepresented in patients with V180I gCJD and P105L GSS [14]. In the four E196A gCJD cases, the PRNP polymorphisms of codon 129 were all 129M/M, which indicates that 129M/M may be susceptible to E196A. However, more cases are needed to test whether E196A is related to the 129M/M polymorphism.

The 14–3-3 protein, NSE, and total tau (non-phosphorylated) proteins in the CSF are less commonly evaluated and are considered less useful markers for genetic prion diseases [1]. The real-time quaking-induced conversion test (RT-QuIC), which detects and amplifies PrP into amyloid fibrils, is considered a more sensitive test than the analysis of CSF biomarkers for the diagnosis of gPrD. The recent development of the second-generation CSF RT-QuIC, which uses shorter fragments of PrP as a substrate, has better diagnostic performance. Further, the combination of PRNP gene sequencing with RT-QuIC allows for the differentiation of major prion subtypes in vivo [15].

The development of precision medicine has allowed for the utilization of genomic information to better understand and treat various diseases. Recently, researchers conducted genomic analyses of five patients with V180I gCJD [16]. Along with V180I mutations in PRNP, genetic variations in other genes, such as lipoprotein(a) (LPA), Leucine-rich repeat kinase 2 (LRRK2), and fibroblast growth factor 20 (FGF20), which are directly or indirectly related to neurodegenerative diseases, were also observed in patients with V180I gCJD [16]. This suggests that the occurrence of gCJD may be affected by other neurodegenerative diseases that are associated with genetic mutations [16]. Parkinson’s disease (PD) is characterized as a movement disorder, and in the current case, the patient presented to our hospital with a movement disorder. Therefore, this patient may carry genetic mutations that are associated with PD. Further genetic analyses should be conducted in the future.

In conclusion, we report a rare case of gCJD in China. The patient carried the glutamate to the alanine substitution at codon 196 (E196A) of PRNP, and provide further knowledge of E196A gCJD. Meanwhile, we discuss the new assistant examination recently perform in the diagnosis and research of gCJD.
